# The Consideration of Future Consequences Scale Among Malaysian Young Adults: A Psychometric Evaluation

**DOI:** 10.3389/fpsyg.2021.770609

**Published:** 2021-12-09

**Authors:** Long She, Lan Ma, Fatemeh Khoshnavay Fomani

**Affiliations:** ^1^Saito University College, Petaling Jaya, Malaysia; ^2^Faculty of Business and Law, Taylor’s University, Subang Jaya, Malaysia; ^3^School of Nursing and Midwifery, Tehran University of Medical Sciences, Tehran, Iran

**Keywords:** consideration of future consequences, Malaysian validation, psychometric evaluation, young adults, factor analysis

## Abstract

**Background:** The consideration of future consequences (CFC) determines the extent to which individuals consider the potential future outcomes of their current behavior. The significance of assessing the CFC scale’s validation in different contexts has been acknowledged by the previous studies. While the majority of the studies have been conducted in western countries, no study has been conducted in Malaysia. The aim of the current study was to validate a Malaysian version of the CFC scale among Malaysian young adults.

**Methods:** The methodological cross-sectional approach was adopted in this study. The study recruited 529 young adults (age range from 25 to 40) who fulfilled the inclusion criteria of the paper survey. Construct validity was assessed using content validity, convergent validity, and discriminant validity. Cronbach’s alpha, McDonald’s omega, and average inter-item correlation (AIC) were used to assess the scale’s internal consistency. Also, composite reliability (CR) and maximal reliability (MaxR) were used to assess the construct reliability. Measurement invariance was tested across gender.

**Results:** The findings of the exploratory factor analysis indicated that the Malaysian version of the CFC scale has a two-factor structure (i.e., CFC-Future and CFC-Immediate) with 10-item explaining 61.682% of the total variance. The confirmatory factor analysis (CFA) supported the two-factor structure of the CFC scale with good construct validity. The internal consistency and CR were acceptable. [The Cronbach’s alpha, McDonald’s omega, and CR for CFC-I were 0.901 (CI 95%: 0.881–918), 0.901, and 0.887, respectively. Also, these parameters for CFC-F were 0.867 (CI 95%: 0.838–891), 0.868, and 0.867, respectively].

**Conclusion:** We found acceptable psychometric evidence for the 10-item two-factors CFC scale used in the context of young adults in Malaysia. The validated instrument can be used in future studies to assess young adults’ CFC tendency and CFC-related behavior in Malaysia.

## Introduction

The consideration of future consequences (CFC) is known as a cognitive construct that reflects the extent to which individuals may consider the immediate and distant outcomes of their current behavior (Strathman et al., 1994; Petrocelli, 2003; Murphy et al., 2020). More specifically, the trade-off between immediate desires and distant future benefits of a potential behavior is thoroughly ruminated while a person is deciding whether to engage in a certain behavior (Rappange et al., 2009). It is used to predict an individual’s act (Vilar et al., 2020) and examine a person’s future awareness (Oliver Schwarz, 2007). Despite the effort, previous studies put into attempting to develop the measurement of this future-oriented cognitive construct, the CFC scale, originally developed by Strathman et al. (1994), has achieved massive attention and satisfying results in terms of internal consistency, construct reliability, and validity. The measurement highlights individual differences in prioritizing the short- or long-term consequences of their behaviors (De Groot and Steg, 2007): some people focus mainly on the immediate benefits of behaviors and are less interested in consequences that may not obtain in the future; others believe that some of their behaviors and followed achievements are as valuable as they can focus on future consequences instead of the immediate outcomes (Strathman et al., 1994). Also, it takes intrapersonal struggle into consideration (Ainin et al., 2015).

Due to CFC’s profound impact on individuals’ current behaviors and attitudes, behavioral studies across various domains such as healthcare, finance, work, and environmental studies have included it in their frameworks (Rappange et al., 2009; Joireman et al., 2012). For instance, the findings of a meta-analysis study reveal that people’s intrapersonal struggle about future consequences is significantly associated with their current health behaviors (Murphy and Dockray, 2018). In addition, the CFC has been associated with motivation, decision-making, goal pursuit, and behavior across other important life domains, including work, health, the environment, finances, and education (Murphy et al., 2020). Study results exhibit the influential positive relationship among CFC with self-prospection and energization (Stephan et al., 2018), key familial relationships (Kataoka and Takizawa, 2019), buffering against aggression related to psychopathy (Zhao et al., 2018), management commitment to safety and safety communication (Mashi et al., 2018), accepting and responding to anthropogenic climate change (Corral-Verdugo et al., 2017), and sustainable consumption (Suárez et al., 2020).

Another application of CFC falls into the validation of the instrument. Strathman et al. (1994) developed the 12-item unidimensional scale to measure individual differences in CFC, the items focused on capturing the extent to which individuals are concerned with the immediate or distant outcomes of their behaviors, and it has gained great popularity. The majority of earlier studies applied it using the average score of all items or the sum of the future items plus the sum of the reverse-coded immediate items (Murphy et al., 2020). The low scores indicate the high priority of the immediate benefits of the behaviors. Conversely, high scorers are expected to prioritize the future implications of their current actions and sacrifice immediate gratification to obtain long-term benefits (Strathman et al., 1994). The validity and the structure of the CFC scale have been widely investigated in the existing literature. For instance, some CFC psychometric studies found the acceptable fit indices for a one-factor model (Crockett et al., 2009; Hevey et al., 2010; Toepoel, 2010). Several studies, however, validated the CFC 12-item scale into a two-factor structure with a combination of CFC-Future (CFC-F) and CFC-Immediate (CFC-I) and demonstrating better fit (Joireman et al., 2008; Rappange et al., 2009; McKay et al., 2016; Vilar et al., 2020). Joireman et al. (2008) argued that the original CFC scale reflects a concern for both distant and immediate consequences of an individual’s action and it is more reliable if the underlying construct is divided into two sub-factors (CFC-F and CFC-I). Moreover, a few studies found that the CFC-F sub-scale in the two-factor models of the CFC scale has shown inadequate reliability coefficients (Petrocelli, 2003; Joireman et al., 2012). Therefore, to provide stronger evidence for the factor structure of bi-dimensional CFC, Joireman et al. (2012) updated the measure by adding two CFC-F items and presented CFC-14. There are still controversial psychometric findings regarding the implementation of CFC-14. Whereas the findings of some confirmatory factor analyses replicated the two-factor model in the CFC-14, supporting the distinction between CFC-Future and CFC-Immediate in domain-specific CFC-14 scales (Nigro et al., 2016; Vásquez-Echeverría et al., 2018; Murphy et al., 2020; Vilar et al., 2020), the other investigations suggested a two-factor model as the best solution for the 13-items (Acuña et al., 2020) or 6-items (Murphy et al., 2020) versions. Nevertheless, Petrocelli (2003) claimed no consistent results among the studies due to the limited sample size. The factor for CFC-Future was unstable and showed poor internal consistency. Thus, the need for further empirical attention to the validation of the 12-item CFC scale had been emphasized in the same study. This study intends to examine a 12-item CFC scale developed by Strathman et al. (1994) to capture how considerate consequences work in a developing country among its young adults with a sufficient sample size to provide further evidence for its validity.

The results of past studies revealed the significance of validating the scale in different contexts as a wide range of studies conducted to investigate the CFC scale psychometric properties are among western developed countries (Rappange et al., 2009; McKay et al., 2016). The findings in non-western population settings are scarce, and little study has been done to support the scale structure validity (Vilar et al., 2020). Therefore, to get valid and generalizable findings, previous psychometric studies suggested that psychology scales should be sensitive to different cultures and valid psychometrically (Yaghoobzadeh et al., 2019; She et al., 2021b).

Malaysia, a multi-ethnic and multi-cultural country, is chosen for the study. First, according to the latest results from the Global Leadership and Organizational Behavior Effectiveness (GLOBE) project, one of the most comprehensive studies assessing cultural practices and differences (House et al., 2004), Malaysian scored relatively high (4.58 out of 7) on future orientation aspect compared with other developed countries as it is a very important aspect of the CFC scale; however, very few studies have been done on assessing the CFC in the Malaysian context, especially for young adults. It may have happened as a result of a lack of validated instruments to investigate the CFC. Also, previous studies have determined that Malaysians tend to view consequences from three perspectives, which include immediate, intermediate, and future consequences (Ainin et al., 2015). As young people tend to have a short span of future awareness, they are more sensitive to their peers and more responsive to the immediate environment. This may lead to compromised decision-making (Steinberg et al., 2008; Bonnie et al., 2015) and result in risky behaviors (Bonnie et al., 2015; She et al., 2021a). This phenomenon has been discovered in previous studies among Malaysian young adults (Pahlevan Sharif and Yeoh, 2018; Pahlevan Sharif et al., 2021) which stated that Malaysian young adults tend to own their possessions and enjoy the current moment, rather than considering the future outcomes of their current behavior, which usually leads to the negative impact on their well-being. To the best of our knowledge, this is the first study on assessing the psychometric properties of the CFC scale among Malaysian young adults. Applying a valid and reliable measure for CFC assessment would help to broaden the possibilities in future studies. Therefore, this study aims to validate the CFC scale to be used in the Malaysian context among young adults.

## Materials and Methods

### Study Design

The data used in this study are part of the broader project on financial well-being among millennials in Malaysia. The methodological cross-sectional approach was adopted in this study. A paper survey was conducted from October 2019 to March 2020 to collect data from young adults in Klang Valley, Johor, and Sabah, Malaysia. The questionnaire survey was carried out in several popular shopping malls and public locations in each region. Potential respondents were first informed about the purpose of the study and the confidentiality of their responses. The inclusion criteria for respondents were Malaysian young adults who (1) aged from 25 to 40 years and (2) showed a willingness to participate in this study. A total of 564 survey forms were retrieved using a convenience sampling technique. In which 529 samples have completed and fulfilled the inclusion criteria of this study.

### Measures

#### Consideration of Future Consequences Scale

The CFC scale is a 12-item unidimensional scale originally developed by Strathman et al. (1994) that aims to measure the individual difference in the extent to which people consider future and immediate consequences of potential behaviors. For this study, participants were asked to rate each item (e.g., “I consider how things might be in the future and try to influence those things with my day-to-day behavior”) using a 10-point Likert scale ranging from 1 (extremely uncharacteristic) to 10 (extremely characteristic). The validity and reliability of the CFC scale have been assessed in different studies and contexts (Petrocelli, 2003; Daugherty and Brase, 2010; Toplak et al., 2011; Joireman et al., 2012; Ainin et al., 2015).

### Procedure

To conduct this study, we first obtained written permission for the use of the CFC scale from the developer of the scale, Professor Alan Strathman, through email. Initially, two versions (English and Malay) of the questionnaire were prepared separately for the broader project on financial well-being to ensure the accuracy of the questionnaire content. Respondents were offered to choose either the English version or the Malay version of the questionnaire. This study only used respondents who responded to the Malay version of the questionnaire to align with the purpose of the psychometric studies. The WHO protocol of forward-backward translation technique was applied for translating the questionnaire from English to Malay. Two English–Malay translators to translate the questionnaire into Malay independently. An expert panel that consists of two professional translators, one professor in linguistics and one expert in psychology, assessed the two translated versions and chose the best statement/wording for each item. Then, the Malay version was sent to a Malay–English translator to back-translate the questionnaire to English. The English version was then sent to an expert in psychology to confirm the correctness of the translation and its equivalence to the original questionnaire. No word in the questionnaire holds different conceptual meanings between original English and targeted (Malay) versions. After getting approval from the expert, the Malay version of the questionnaire was used for data gathering.

### Content Validity

To assess the content validity of the Malaysian CFC scale, we used content validity ratio (CVR) and modified kappa coefficient (K) to ensure the instrument fully assesses or measures the construct of interest. To obtain the CVR and modified kappa coefficient (K), we provided the 12-item CFC scale to 10 experts in the field of psychology, health, and business to seek comments on the wording, item allocation, and scaling of the items. Subsequently, they were required to respond to the essentials of the CFC scale items using the following: not essential, useful but not essential, and essential. The value of CVR then was calculated using the formula of [*ne* – (*N*/2)]/(*N*/2), where *ne* is the number of experts who rate the item as “essential” and *N* is the total number of the experts (Cook and Beckman, 2006). According to Lawshe (1975), the accepted value for CVR should be greater than 0.62 when the number of experts is 10 (Lawshe, 1975). Next, the modified kappa coefficient (K) of each item was calculated, and a value greater than 0.6 is considered good (Wynd et al., 2003).

### Construct Validity

To assess the construct validity and reliability, we randomly divided the dataset (*n* = 529) into two sub-samples using the Excel RAND function: the first 265 cases were used as the first sub-sample and the remaining 264 cases were used as second sub-sample. This study first conducted the maximum likelihood exploratory factors analysis (EFA) with Promax rotation using SPSS version 26 using the first dataset (*n* = 265). The Kaiser–Meyer–Olkin (KMO) test and Bartlett’s test of sphericity were used to check the adequacy of the sampling and appropriateness of the data for factor analysis. Hair et al. (2010) suggested that KMO values greater than 0.5 are considered acceptable for factor analysis. Also, a *p-*value of Bartlett’s test of sphericity less than 0.05 indicates the adequacy of the sampling. The following criteria were used to extract the factor structure: (1) eigenvalues > 1; (2) communalities > 0.3, and (3) scree plots (Cattell, 1966; Cattell and Jaspers, 1967; Field, 2013). Also, items with a factor loading of more than 0.5 were considered acceptable in this study (Kamadi et al., 2016; She et al., 2021b). Next, using the second dataset (*n* = 264), the maximum likelihood confirmatory factor analysis (CFA) was conducted using AMOS version 27 to confirm and validate the factor structure obtained from EFA. The model fit was assessed according to several fit indices such as chi-square (χ^2^) test, chi-square/degree of freedom ratio (χ^2^/*df*) < 4, comparative fit index (CFI) > 0.90, Tucker–Lewis index (TLI) > 0.90, root mean square error of approximation (RMSEA) < 0.08, and standardized root mean square residual (SRMR) < 0.09 (Pahlevan Sharif and Sharif Nia, 2018). Moreover, convergent and discriminant validity were used to assess the construct validity. To establish good convergent validity, composite reliability (CR) should be greater than 0.7, average variance extracted (AVE) of each construct should be greater than 0.5 and less than its respect CR (Sharif et al., 2019; She et al., 2021b). For discriminant validity, this study followed the two methods, namely, the Fornell–Larcker criterion and the heterotrait-monotrait ratio of correlations (HTMT) anaysis. To meet the requirements, the square root of the AVE of each construct should be greater than its correlation with other constructs (Fornell and Larcker, 1981), and all values of the HTMT matrix should be less than 0.85 (Henseler et al., 2015).

### Reliability Assessment

The reliability of the Malaysian version of the CFC scale was evaluated through its internal consistency [Cronbach’s alpha, McDonald’s omega, and average inter-item correlation (AIC)], CR, and maximal reliability (MaxR). To achieve good internal consistency and reliability, the Cronbach’s alpha, McDonald’s omega, CR, and MaxR for each construct should be greater than 0.7 (Hair et al., 2014; Sharif et al., 2019).

### Measurement Invariance for Gender

Measurement invariance was tested to assess whether the measurement model of the Malaysian version of the CFC scale could be held for both male and female groups. Four nested models were established to test configural invariance, metric invariance, scalar invariance, and residual invariance (Putnick and Bornstein, 2016). Invariance was assumed using the absolute value of ΔCFI < 0.01 and ΔRMSEA < 0.01 criteria (Cheung and Rensvold, 2002). Also, Δchi-square (Δχ^2^) and its significant level were used to further examine the evidence of the measurement invariance.

### Multivariate Normality and Outliers

This study also tested the univariate and multivariate distributions. The outliers and the values of the skewness and kurtosis were identified through the testing of univariate distributions. Moreover, for multivariate distributions, multivariate normality was tested using Mardia’s coefficient of multivariate kurtosis of more than 8 (Raoprasert and Islam, 2010). Also, Mahalanobis distance was used to identify the multivariate outliers where items with a Mahalanobis distance of *p* < 0.001 were deemed as multivariate outliers (Harrington, 2009).

### Ethical Considerations

The Ethics Committee of Taylor’s University, Malaysia, approved the Ethical Considerations of this study (reference no.: HEC 2019/073). In addition, all participants were informed of the data collection purpose, and questionnaires were distributed to the respondents only after they provided their consent to participate in the survey. Moreover, the respondents were ensured that their participation was on the voluntary basis, and the confidentiality of all collected data was guaranteed.

## Results

### Characteristics of the Respondents

In total, 529 young adults completed the questionnaire. As shown in Table 1, the sample of this study consisted of 242 (45.7%) men and 287 women (54.3%) with a mean age of 31.97 (*SD* = 4.77) years. Most participants were Malay (57.4%), married (53.5%), and had a full-time job (81.1%). With respect to the level of education, 62.6% of the total respondents held at least a bachelor’s degree, as for the income level of the respondents, 79.8% of them earned incomes below RM5,000 per month.

**TABLE 1:** The characteristics of the respondents.

Variable		Frequency (*N*)	Percentage (%)
Gender			
	Male	242	45.7
	Female	287	54.3
Ethnicity			
	Malay	304	57.4
	Chinese	121	22.9
	Indian	31	5.9
	Others	73	13.8
Marital Status			
	Single	227	42.9
	Married	283	53.5
	Divorced	13	2.5
	Widowed	6	1.1
Education			
	Secondary school and below	73	13.8
	Diploma	122	23.1
	Bachelor’s Degree	194	36.7
	Master’s Degree	98	18.5
	Doctoral Degree	39	7.4
	Others	3	0.6
Employment Status			
	Never worked	9	1.9
	Unemployed	35	6.6
	Part-time employment	55	10.4
	Full-time employment	430	81.1
Monthly Income			
	Less than RM2,000	126	23.8
	RM2,001 to RM3,000	147	27.8
	RM3,001 to RM5,000	149	28.2
	RM5,001 to RM7,000	62	11.7
	RM7,001 to RM9,000	29	5.5
	Above RM9,000	16	3.0

### Content Validity

Content validity of the CFC scale was evaluated using CVR and modified kappa coefficient (K). The results showed that the CVR of all 12 items was greater than the minimum value of 0.62 suggested by Lawshe (1975). Also, the value of the modified kappa coefficient (K) for all 12 items of the CFC scale was greater than 0.6. Thus, no item was excluded in this stage.

### Construct Validity

The results of maximum likelihood EFA with Promax rotation (*n* = 265) on the Malaysian version of the CFC scale are reported in Table 2. The KMO value was 0.859, and the results of Bartlett’s test of sphericity were significant (*p* < 0.001, 1152.818, *df* = 45), which indicates the adequacy of the sampling and appropriate for the factor analysis. Moreover, two factors were extracted from EFA, explaining 61.682% of the total variance of the sample. Two items (item 2 and item 5) were removed because of weak factor loading of less than 0.5. Referring to the items related to each sub-factor, the two factors were named (1) consideration of immediate consequences (CFC-I) and (2) CFC-F.

**TABLE 2:** The result of EFA on the two-factor Malaysian version of CFC scale.

Factor	Items	Factor loading	Communalities	Eigenvalues	% Variance
Consideration of immediate consequences	**3**. I only act to satisfy immediate concerns, figuring the future will take care of itself.	0.574	0.354	4.058	40.581
	**4**. My behavior is only influenced by the immediate (i.e., a matter of days or weeks) outcomes of my actions.	0.517	0.387		
	**9**. I generally ignore warnings about possible future problems because I think the problems will be resolved before they reach crisis level.	0.679	0.452		
	**10**. I think that sacrificing now is usually unnecessary since future outcomes can be dealt with at a later time.	0.894	0.751		
	**11.** I only act to satisfy immediate concerns, figuring that I will take care of future problems that may occur at a later date.	0.884	0.750		
	**12.** Since my day-to-day work has specific outcomes, it is more important to me than behavior that has distant outcomes.	0.743	0.595		
Consideration of future consequences	**1.** I consider how things might be in the future, and try to influence those things with my day-to-day behavior.	0.645	0.412	2.110	21.101
	**6.** I am willing to sacrifice my immediate happiness or well-being in order to achieve future outcomes.	0.663	0.514		
	**7**. I think it is important to take warnings about negative outcomes seriously even if the negative outcome will not occur for many years.	0.809	0.626		
	**8**. I think it is more important to perform a behavior with important distant consequences than a behavior with less-important immediate consequences.	0.635	0.398		

Subsequently, the maximum likelihood CFA was conducted (*n* = 264) to confirm and validate the factor structure obtained from EFA. Given that the factor structure obtained from EFA consisted of two latent variables with 10 items. The Web-macro named “Computing Power and Minimum Sample Size for RMSEA” was used to calculate the minimum sample size required for CFA model-based alpha level, degrees of freedom, desired power, null RMSEA level, and alternative RMSEA level (Preacher and Coffman, 2006, Web-macro available online: http://www.quantpsy.org/rmsea/rmsea.htm). The CFA model would have 34 degrees of freedom based on the following equation: number of items × (number of items + 1)/2 – 2 × number of items – number of latent variables × (number of latent variables)/2 (Rigdon, 1994). Using 34 degrees of freedom accompanied by type 1 error at 0.05 (alpha), desired power at 0.8, null RMSEA at 0, and alternative RMSEA at 0.08, the minimum sample size required for achieving desired power is 120. Therefore, 264 samples were sufficient to provide adequate statistical power in the study.

**FIGURE 1:**
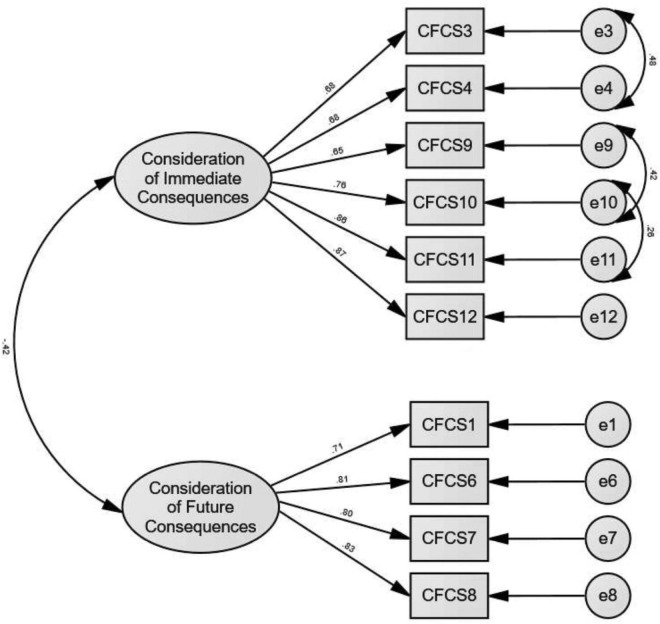
The final measurement model of CFC scale among Malaysian young adults.

The results of CFA showed that the initial two-factor measurement model did not achieve good model fit as evidenced by {χ^2^ (34) = 186.750, *p* < 0.05, χ^2^/*df* = 5.493, CFI = 0.902, TLI = 0.870, SRMR = 0.077, and RMSEA (90% CI) = 0.131 [0.113, 0.149]}. After reviewing the model modification indices, three pairs of the measurement error were allowed to covary (between item 3 and item 4, item 9 and item 10, and item 10 and item 11). The results showed that all the factor loadings were higher than 0.5 and significant (Figure 1).

Also, the revised measurement model showed a good model fit {χ^2^ (31) = 81.026, *p* < 0.05, χ^2^/*df* = 2.614, CFI = 0.968, TLI = 0.953, SRMR = 0.067, RMSEA (90% CI) = 0.078 [0.058, 0.099]} and improved significant as evidence by a significant reduction in chi-square (Δ*x*^2^ = 105.72, Δ*df* = 3, *p* < 0.01). Moreover, the results indicated that AVE for both factors was greater than 0.5 and less than its respective CR (Table 3); therefore, a good convergent validity was achieved. Also, Table 4 shows that the square root of AVE of each construct was greater than the absolute value of the correlation with other constructs (Fornell and Larcker, 1981), and the value in the HTMT matrix was less than 0.85, which indicates that the good discriminant validity was achieved (Henseler et al., 2015).

**TABLE 3:** Construct reliability and convergent validity of Malaysian version of CFC scale (*N* = 264).

Factors	Cronbach’s alpha (CI 95%)	McDonald’s omega	AIC*	CR**	MaxR***	AVE****
Consideration of immediate consequences	0.901 (0.881, 0.918)	0.901	0.528	0.887	0.907	0.569
Consideration of future consequences	0.867 (0.838, 891)	0.868	0.475	0.867	0.873	0.621

*AIC: average inter-item correlation.

**CR: composite reliability.

***MaxR: maximal reliability.

****AVE: average variance extracted.

**TABLE 4:** Discriminant validity assessment using the Fornell–Larcker criterion and HTMT matrix.

	Construct	Consideration of immediate consequences	Consideration of future consequences
Fornell–Larcker criterion	Consideration of immediate consequences	0.754	
	Consideration of future consequences	–0.418	0.788
Heterotrait-monotrait ratio of correlations (HTMT)	Consideration of immediate consequences		
	Consideration of future consequences	0.417	

### Reliability

As shown in Table 3, both factors achieved good internal consistency and construct reliability, as evidenced by Cronbach’s alpha, McDonald’s omega, CR, and MaxR. The Cronbach’s alpha, McDonald’s omega, CR, and MaxR for CFC-I were 0.901 (CI 95%: 0.881–918), 0.901, 0.887, and 907, respectively. Also, the Cronbach’s alpha, McDonald’s omega, CR, and MaxR for CFC-F were 0.867 (CI 95%: 0.838–891), 0.868, 0.867, and 873, respectively. Moreover, the AIC values of both factors were good (CFC-F: 0.528; CFC-I: 0.475).

### Gender Invariance

Table 5 shows that the results for the analysis of gender invariance of the Malaysian version of CFC scale, using the absolute value of ΔCFI < 0.01 and ΔRMSEA < 0.01 criteria, metric invariance (ΔCFI = –0.003 and ΔRMSEA = –0.001), scalar invariance (ΔCFI = –0.001 and ΔRMSEA = –0.002), and residual invariance (ΔCFI = 0 and ΔRMSEA = –0.003) were all found between male and female groups. Also, the results showed that both scalar invariance [Δχ^2^_scalar_ (8) = 11.814, *p* = 0.160] and residual invariance [Δχ^2^_residual_ (10) = 9.326, *p* = 0.501] were confirmed between male and female groups based on criterion non-significant of Δχ^2^ criterion; however, the metric invariance was not confirmed under this criterion [Δχ^2^_metric_ (8) = 15.665, *p* = 0.047 < 0.05].

**TABLE 5:** Gender invariance analysis of Malaysian version of CFC scale.

Model	Chi-square	*df*	CFI*	TLI**	RMSEA***	SRMR****	Δchi-square	Δ*df*	Sig.	ΔCFI	ΔRMSEA
Configural invariance	149.948	62	0.967	0.952	0.052	0.0586	-	-	-	-	-
Metric invariance	165.613	70	0.964	0.954	0.051	0.0644	15.665	8	0.047	–0.003	–0.001
Scalar invariance	177.427	78	0.963	0.957	0.049	0.0644	11.814	8	0.160	–0.001	–0.002
Residual invariance	186.753	88	0.963	0.962	0.46	0.0693	9.326	10	0.501	0	–0.003

*Comparative fit index (CFI).

**Tucker–Lewis index (TLI).

***Root mean square error of approximation (RMSEA).

****Standardized root mean square residual (SRMR).

## Discussion

The aim of this study was to validate the CFC scale in the Malaysian context among young adults. The EFA results showed a 10-item bi-dimensional construct, while the CFA results confirmed the two-factor version of the CFC scale, which showed a goodness-of-fit. As a result, the final version of the Malaysian CFC scale showed good internal consistency and construct reliability and validity. The findings of the study supported the factorial validity of the two-factor model of the CFC scale that have conducted in Portugal (Echeverría et al., 2015), Italy (Hevey et al., 2010; Nigro et al., 2016), and Netherlands (Joireman et al., 2012).

The results of the study revealed that the Malaysian version of the CFC scale consists of ten items compared with the 12-item structure of the original CFC scale. Item 2 and item 5 were removed due to weak loadings of less than 0.5. These findings were in line with Vilar et al. (2020)’s study on university students from Brazil and New Zealand. Similarly, item 5 *(my convenience is a big factor in the decisions I make or the action I take)* in which exhibited poor factor loading has been found in a study conducted in the Spanish and Portuguese context (Vásquez et al., 2013; Echeverría et al., 2015). The possible explanation can be that a mixture of decision-making and action-taking activities in the same item confuses participants (Vilar et al., 2020). Also, item 2 was dropped in a study conducted among Dutch people (Rappange et al., 2009). According to Vilar et al. (2020), item 2 (*often I engage in a particular behavior in order to achieve outcomes that may not result for many years*) implicitly indicates the length of time and cause complexity in understanding.

The findings of the current study indicated that the initial two-factor measurement model did not achieve a good model fit. Therefore, further investigation was carried out to find out possible misspecifications and significantly correlated measurement errors that can be suggested as a plausible reason for inappropriate initial model fit. The findings revealed that three pairs of items error were covaried (item 3 and item 4, item 9 and item 10, and item 10 and item 11). Non-random measurement errors may correlate with the assessment method of the self-administered questionnaires that results in some common response biases related to the self-reporting method (Brown, 2015). Correlated measurement error, therefore, can be used as a scale reduction technique to finalize and formulate the final version of the scale’s structure (Floyd and Widaman, 1995).

The high level of CR, Cronbach’s alpha, McDonald’s omega, and the correlation between the items demonstrated that the revised two-factor structural of the scale had good internal consistency and reliability. The current findings were in accordance with the previous studies that indicated the same results across different contexts (Echeverría et al., 2015; Murphy et al., 2020; Vilar et al., 2020). A longitudinal study (from 1996 to 2006) with a sample of Dutch-speaking population in Netherlands demonstrated that the CFC scale was internally very consistent over time. Considering the aforementioned result, the researchers suggested that the kind of sample recruited in the study might be a better indicator of the CFC scale consistency than the measure itself (Toepoel, 2010).

Although the original 12-item one-factor CFC scale developed and tested by Strathman et al. (1994) accounted for 94.6% of the variance, the study by Vilar et al. (2020) indicated that the two-factor refined version of the CFC scale demonstrating better fit and explaining the total variance of 28% for CFC-F and 33% of CFC-I. This study showed that the Malaysian version of the two-factor structure of the CFC scale explained a satisfactory level of the total variance (61.68%) compared with previous studies, where CFF-I (40.58%) showed a higher level of total variance explained compared with CFC-F (21.10%). This is supported the notion that young people are more regulated by the concern with the immediate consequences of the behavior than the future (Bonnie et al., 2015; Pahlevan Sharif and Yeoh, 2018).

The current study indicated that the two-factors CFC scale has adequate convergent validity as supported by the higher level of AVE and CR. Despite the fact that most previous studies have tested the CFC scale’s convergent validity by providing empirical shreds of evidence for the relationship between the CFC scale and some other measures such as “Willingness to delay gratification and locus of control” (Toepoel, 2010), this study findings support the previous studies regarding the adequate convergent validity of the CFC scale (Nigro et al., 2016; Vilar et al., 2020).

This study was conducted in response to the lack of a specific measurement tool for assessing the extent to which young adults consider the potential future outcomes of their current behavior. Considering that young people tend to have a short span of future awareness, they are more vulnerable to being involved in risky behaviors that affect their health status (Steinberg et al., 2008; Bonnie et al., 2015). The validated Malaysian version of the CFC is applicable for health workers and policymakers to develop health promotion programs according to accurate data.

## Study Limitations

This study, however, is not without limitations. First, the study applied a convenient sampling method from the selected geographical urban region in Malaysia. Non-probabilistic samples were enrolled in this study restrict the generalizability of the findings. As such findings may not be broadly generalizable to the rural population, future studies are suggested to collect data from a more representative sample using other sampling methods. Second, using the self-report measure of the instrument, the construct of consideration for the future consequences might be affected by the participants’ social desirability. As a result, the participants might mask their actual psychological functioning (Vilar et al., 2020). Moreover, due to cultural differences, language nuances may not be fully translated. Thus, further studies are needed to test the validity and reliability of this construct across the various populations and contexts.

## Conclusion

This study provides the first validation of the CFS scale among Malaysian young adults. We found acceptable psychometric evidence for the 10-item two-factors CFC scale used in the context of young adults in Malaysia. Therefore, the validated instrument can be used in future studies to access young adults’ CFC tendency and CFC-related behavior in Malaysia.

## Data Availability Statement

The raw data supporting the conclusions of this article will be made available by the authors, without undue reservation.

## Ethics Statement

The studies involving human participants were reviewed and approved by The Ethics Committee of Taylor’s University, Malaysia; Reference No. HEC 2019/073. The patients/participants provided their written informed consent to participate in this study.

## Author Contributions

LS and LM: study conception and design. LS: data collection, analysis, and interpretation of results. FK, LS, and LM: draft manuscript preparation. All authors reviewed the results and approved the final version of the manuscript.

## Conflict of Interest

The authors declare that the research was conducted in the absence of any commercial or financial relationships that could be construed as a potential conflict of interest.

## Publisher’s Note

All claims expressed in this article are solely those of the authors and do not necessarily represent those of their affiliated organizations, or those of the publisher, the editors and the reviewers. Any product that may be evaluated in this article, or claim that may be made by its manufacturer, is not guaranteed or endorsed by the publisher.
